# Problematic Gambling Behavior in a Sample of Gamblers: The Role of Alexithymia, Dissociation Features, and External Locus of Control

**DOI:** 10.1007/s10899-024-10322-6

**Published:** 2024-06-04

**Authors:** Alessio Gori, Eleonora Topino

**Affiliations:** 1 Department of Health Sciences, University of Florence, Via di San Salvi 12, Pad. 26, Firenze, 50135 Italy; 2 Integrated Psychodynamic Psychotherapy Institute (IPPI), Via Ricasoli 32, Florence, 50122 Italy; 3 Department of Human Sciences, LUMSA University of Rome, Via della Traspontina 21, Rome, 00193 Italy

**Keywords:** Absorption, Alexithymia, Behavioural addictions, Dissociation, Gambling disorder, Locus of control

## Abstract

Gambling disorder is a severe condition that significantly compromises the lives of those affected. In light of this clinical relevance, the literature on the antecedents of this form of addiction is constant and continually evolving. Consistently with this framework, the present research aimed at investigating the role of some risk factors in gambling disorder, with a specific focus on alexithymia, dissociation, and locus of control. The research involved a sample of 290 participants (183 males and 107 females; M*age* = 34.43, *SD* = 14.65) who practice gambling at least occasionally. They completed an online survey including the South Oaks Gambling Screen, Twenty-Items Toronto Alexithymia Scale, Dissociative Experience Scale‐II, and Locus of Control of Behavior. ANOVA and a moderated-mediation model were implemented to analyse the collected data. Results showed that 19.0% of the participants were At Risk and Problem Gamblers, while 27.6% fell into the category of Problematic Gamblers. Problematic Gamblers showed significantly higher levels of alexithymia, dissociation, and external locus of control. Moreover, a significant association between alexithymia and the severity of problematic gambling behaviour was found and was significantly mediated by absorption (a dissociation feature). Furthermore, the external locus of control significantly moderated this indirect effect. The role of gender as a covariate was also investigated. Such findings may offer further insights into the field of clinical research on gambling disorder and may provide useful information for effective clinical practice.

**Supplementary Information:**

The online version contains supplementary material available at 10.1007/s10899-024-10322-6.

## Introduction

For certain individuals, known as social gamblers, gambling can be a leisure activity, i.e., a relaxing pastime that entails no negative consequences (Temcheff et al., 2016). However, for others, this activity can become problematic to varying degrees, sometimes leading to the development of an addiction. Gambling disorder, in fact, has been classified within the category of “*Substance-Related and Addictive Disorders*” since the fifth edition of the Diagnostic and Statistical Manual of Mental Disorders (DSM-5; American Psychiatric Association [APA], 2013, 2022), referring to persistent and excessive gambling behaviour that causes clinically significant distress. In fact, this condition has been associated with numerous psychosocial issues, such as family and interpersonal problems (Shaw et al., 2007) and emotional difficulties (Rogier et al., 2021; Jauregui et al., 2016). Moreover, individuals with gambling disorder often face financial, employment, and legal challenges (Welte et al., 2016), along with elevated rates of suicidal ideation, suicide attempts, and completed suicides (Black et al., 2015; Thon et al., 2014). Given the clinical significance of this condition, the scientific community consistently maintains a keen and ongoing interest in studying factors that may contribute to a greater vulnerability to this type of behavioural addiction (see Dowling et al., 2017, for a review). This approach seeks to offer direction and guidance for clinical and preventive efforts. Aligning with this, the present research aimed at investigating the role of some risk factors in gambling disorder, with a specific focus on alexithymia, dissociation, and locus of control.

### Alexithymia and Gambling Disorder

Among the various risk factors for the potential development of a gambling disorder, previous research has emphasized the significant role of the inadequate management of emotional experiences (Rogier & Velotti, 2018). In light of this, the scientific literature has specifically focused on alexithymia (see Marchetti et al., 2019, for a review). Alexithymia is characterized by a diminished ability to identify and articulate feelings, difficulty in differentiating emotions from bodily sensations, restricted imaginative processes, and impaired introspective thinking (Taylor et al., 1991; Taylor, 2000). Individuals with alexithymia may try to regulate their emotions through compulsive behaviours: the inability to modulate and tolerate negative affects can drive them to seek emotional regulation through external objects and behavioural actions (Taylor et al., 1991; Caretti et al., 2018; Pellerone et al., 2017). Indeed, alexithymia is frequently observed in individuals with Gambling Disorder (Mestre-Bach et al., 2023) and it may be associated not only with vulnerability (Gori et al., 2022a, 2023a), but also with the severity of the disorder (Gori et al., 2023b; Noël et al., 2018), and the symptoms associated with it, such as increased loss chasing (Bibby & Ross, 2017) or feelings of hopelessness (Estévez et al., 2023), to name a few.

### The Mediation of Dissociation

Within the extensive array of psychopathological variables potentially linked to gambling disorder, dissociation also deserves significant attention. Dissociation is a multidimensional construct that can represent a normal process allowing individuals to protect themselves from overwhelming experiences. It involves a temporary disengagement from reality and the compartmentalization of behaviours, feelings, thoughts, and memories associated with distressing experiences (Loewenstein, 2022). However, when this becomes the individual’s primary response as a means to escape from a painful reality, this strategy becomes maladaptive since prevents adequate affective processing and management, thereby increasing the risk of psychopathology (Guglielmucci et al., 2019). Consistently, previous research finds a significant positive association between dissociation and gambling disorder (see Rogier et al., 2021 for a meta-analysis). Furthermore, dissociation has been shown to play a mediating role in the relationship between alexithymia and gambling behaviours (Topino et al., 2021, 2023). Despite these findings, evidence regarding the distinctive contribution of dissociative subdimensions remains limited (Rogier et al., 2022), necessitating further studies in this domain (Rogier et al., 2021).

### The Role of External Locus of Control

The locus of control (LOC) refers to the extent to which individuals believe that their actions or abilities influence the events in their lives, as opposed to external factors like chance, luck, or other uncontrollable circumstances in the environment (Rotter, 1966). Individuals with a high internal locus of control attribute their outcomes to their internal abilities and resources, while those with a high external locus of control are more inclined to believe that events occur due to factors beyond their control, such as luck or destiny (Wallston, 2001). LOC seems to play a significant role in influencing mental well-being (Mirowsky & Ross, 2003). While research investigating the link between this variable and gambling is limited, the available evidence suggests that individuals higher external locus of control may be more susceptible to engaging in excessive gambling behaviour (Thrasher et al., 2011; Zhou et al., 2012). Therefore, it is conceivable that this variable may somehow facilitate pathways that could increase the risk of developing a gambling disorder, and more research is needed to clarify this aspect.

### The Present Research

Given the aforementioned framework, the present research specifically aimed at:

- Investigating the differences in the levels of alexithymia, dissociation, and locus of control based on gambling severity, to enhance comprehension of the features associated with the severity of problematic gambling behaviour.
- Testing the relationship between alexithymia, dissociation subdimensions, and locus of control in contributing to the severity of problematic gambling behaviour.

Based on the existing scientific evidence, it was expected to find significantly higher levels of alexithymia, dissociation, and external locus of control in gamblers with higher severity of problematic gambling behaviour (**H1**).

Furthermore, to fulfil the second objective, a moderated-mediation model was tested, hypothesizing that: alexithymia would be associated with the severity of problematic gambling behaviour (**H2**); the dissociation subdimensions would mediate the relationship between alexithymia and severity of problematic gambling behaviour (**H3**); external locus of control would moderate the relationship between dissociation and severity of problematic gambling behaviour (**H4**). Finally, since previous studies have shown gender differences for the variables involved in the model (De Pasquale et al., 2018; Rueda Ruiz et al., 2023), this factor was controlled as a covariate to test the solidity of the hypothesized interactions.

## Method

### Participants, Procedure and Ethics

A cross-sectional design was adopted for this research. Participants were recruited through an online snowball procedure and completed a survey hosted on the Google Form platform. Inclusion criteria were having a good command of the Italian language and declaring having gambled at least once in the last year. A sample of 290 participants who practice gambling at least occasionally was involved in this research. The majority of them declared they had a high school diploma (44.5%) and worked as an employee (41.7%). Regarding their marital status, most were single (64.8%), followed by those who were married (17.2%) and cohabiting (10.7%). Before starting the administration, each participant was informed of the research’s general objectives and provided electronic informed consent. The study protocol received approval from the institutional Ethics Committee of the Integrated Psychodynamic Psychotherapy Institute (IPPI; ethical approval number 008/2023).

### Measures

*South Oaks Gambling Screen* (SOGS). The SOGS is a self-report scale used to evaluate the levels of problematic gambling (Lesieur & Blume, 1987; Italian version: Guerreschi & Gander, 2000). The scale includes 16 items rated with different response formats. Some of these items are purely informative for the clinician and are not included in the final score (e.g., the first question on a three-point scale “*not at all*”; “*Less than once a week*”; “*Once a week or more*”). Others are in multiple-choice format, and only one point is assigned for a specific response indicated on the scoring sheet (i.e., items 4, 5, and 6). Finally, other questions are in a yes/no response format, and one point is assigned for a “yes” response. The total score on the South Oaks Gambling Screen (SOGS) categorizes individuals into three types of gambling behaviour: Absence of Gambling Problems (Scores ranging from 0 to 2); At-risk for Problematic Gambling (Scores ranging from 3 to 4); Problematic Gambling (Scores of 5 or more). The Italian version showed good internal consistency in the present sample (α = 0.94).

*Twenty-Items Toronto Alexithymia Scale* (TAS‐20). The TAS‐20 is a self‐report scale used to evaluate the levels of alexithymia (Bagby et al., 1994; Bagby, Taylor, & ParkeBagby et al., 1994a, b; Italian version: Bressi et al., 1996). The scale includes 20 items scored on a 5‐point Likert scale (from 1 = “*strongly disagree*” to 5 = “st*rongly agree*”). Both a total score and three subscales (difficulty identifying feelings; difficulty describing feelings; externally oriented thinking) may be calculated. The total score of the Italian version showed good internal consistency in the present sample (α = 0.85).

*Dissociative Experience Scale-II* (DES‐II). The DES‐II is a self‐report scale used to evaluate the levels of dissociative experiences (Carlson & Putnam, 1993; Italian version: Schimmenti, 2016). The scale includes 28 items scored on an 11‐point scale (from 0% = “*never*,” to 100% = “*always*”). Both a total score and three subscales (dissociative amnesia; absorption; depersonalization‐derealization) may be calculated. The Italian version showed good internal consistency in the present sample (total score, α = 0.96; dissociative amnesia, α = 0.90; absorption, α = 0.88; depersonalization‐derealization, α = 0.92).

*Locus of Control of Behavior* (LCB). The LCB is a self-report scale used to evaluate the locus of control (Craig et al., 1984; Italian version: Farma, & Cortivonis, 2000). The scale includes 17 items scored on a 6-point scale (from 0 = “*strongly disagree*” to 5 = “st*rongly agree*”). Two subscales (Internal Locus of Control Behaviors; External Locus of Control Behaviors) may be calculated. The Italian version showed good internal consistency in the present sample (Internal Locus of Control Behaviors, α = 0.78; External Locus of Control Behaviors, α = 0.86).

### Data Analysis

The collected data were analysed with the SPSS software (version 21.0; IBM, Armonk, NY, USA) for Windows. First, descriptive statistics were calculated. ANOVA was conducted to investigate variations in the levels of alexithymia, dissociation, and internal/external locus of control (dependent variables) in relation to the severity of problematic gambling behaviour. The levels of Gambling Disease (Absence of Gambling Disease; At Risk for Gambling Disease; Problematic Gambling) were included as independent variables. A Bonferroni-adjusted *p-*value of 0.012 was set as the criterion of significance. Post-hoc analyses were performed using the Scheffé test to enhance the interpretation of observed differences. Thenthe parallel mediation of the dissociation subdimensions (dissociative amnesia, depersonalization-derealization, and absorption) in the relationship between alexithymia and severity of problematic gambling behaviour with the moderation of external locus of control was explored with the macro-programm PROCESS v. 3.4 (Hayes, 2018; model 14). The effect of gender as a covariate was also investigated (men coded as 0 and women coded as 1). The Johnson–Neyman (Johnson, & Neyman, 1936) procedure was used to assess the conditional indirect effects of alexithymia on the severity of problematic gambling behaviour through the dissociation subdimensions at the three levels of external locus of control (-1 SD mean, + 1 SD). The *R*^*2*^ index was also explored to detail the interpretation of the final mediation model, by considering Cohen’s thresholds: *R*^*2*^ < 0.02 = very weak effect; 0.02–0.12 = weak effect; 0.13–0.26 = moderate effect; *R*^*2*^ > 0.26 = substantial effect (Cohen, 1988). The bootstrapping method (5000 bootstrapped samples with 90% CI) was finally implemented to support the statistical stability of the model, supporting the significance of the effect when the bootstrapped confidence interval (from boot LLCI to boot ULCI) does not contain zero (Preacher & Hayes, 2008).

### Results

#### Descriptive Statistics of the Sample

As shown in Table 1, participants (183 males and 107 females) had a mean age of 34.43 years (*SD* = 14.65). The majority of them declared they had a high school diploma (44.5%) and worked as an employee (41.7%). Regarding their marital status, most were single (64.8%), followed by those who were married (17.2%) and cohabiting (10.7%). The majority of participants (53.4%) showed an Absence of Gambling Disease according to the SOGS scores, while 19.0% are identified as At Risk and Problem Gamblers, and 27.6% fall into the category of Pathological Gamblers (see Table 1).

**Table 1:** Descriptive Statistics of the sample (*N =* 290)

Characteristics		M ± SD	*n*	%
*Age* (years)		34.4 ± 14.65		
*Sex*				
	Females		183	63.1
	Males		107	36.9
*Marital Status*				
	Single		183	63.1
	Married		50	17.2
	Cohabiting		31	10.7
	Separated		7	2.4
	Divorced		8	2.8
	Widowed		6	2.1
*Education*				
	Middle School diploma		29	10.0
	High School diploma		129	44.5
	University degree		74	25.5
	Master’s degree		47	16.2
	Post-lauream specialization		11	3.8
*Occupation*				
	Student		68	23.4
	Working student		44	15.2
	Employee		121	41.7
	Freelance		6	2.1
	Entrepreneur		7	2.4
	Artisan		15	5.2
	Trader		1	0.3
	Homemaker		4	1.4
	Unemployed		13	4.5
	Retired		11	3.8
*Severity of gambling-related problems (SOGS)*				
	Absence of Gambling Disease		155	53.4
	At Risk for Gambling Disease		55	19.0
	Problematic Gambling		80	26.7

### Differences Based on Gambling Severity

The results of the ANOVA highlighted statistically significant differences in the levels of alexithymia (*F*_2,287_ = 56.022, *p* < 0.001), dissociation (*F*_2,287_ = 142.315, *p* < 0.001), and external LOC (*F*_2,287_ = 145.365, *p* < 0.001) based on the levels of Gambling Disease. Specifically, as the severity of problematic gambling behaviour increased, subjects exhibited higher levels of alexithymia, dissociation, and external LOC (see Table 2). On the other hand, no significant differences, were identified regarding the internal Locus of Control (LOC): *F*_2,287_ = 3.942, *p* = 0.020.

**Table 2:** Means, standard deviation and comparisons of Alexithymia, Dissociation, and Locus of Control Behaviours (LOC; external and internal) based on the levels of Gambling Disease

	Absence		At Risk		Problematic		F	*p*	Scheffé Post hoc
	(*N* = 155)		(*N* = 55)		(*N* = 80)				
	M	SD	M	SD	M	SD			
Alexithymia	39.477	9.921	47.582	9.867	54.125	11.078	56.022	**< 0.001**	P < A, R A < R
Dissociation	12.355	8.655	28.487	12.693	40.550	17.422	142.315	**< 0.001**	P < A, R A < R
External LOC	6.658	5.736	15.945	7.049	24.288	10.623	145.365	**< 0.001**	P < A, R A < R
Internal LOC	23.374	6.375	20.818	5.423	22.900	4.890	3.942	0.020	-

*Note* Bold values indicate *p* within the criteria of significance (Bonferroni - adjusted *p* < 0.012); P = Problematic Gambling; R = At Risk for Gambling Disease; A = Absence of Gambling Disease

### Moderated Mediation Model

**Fig. 1:**
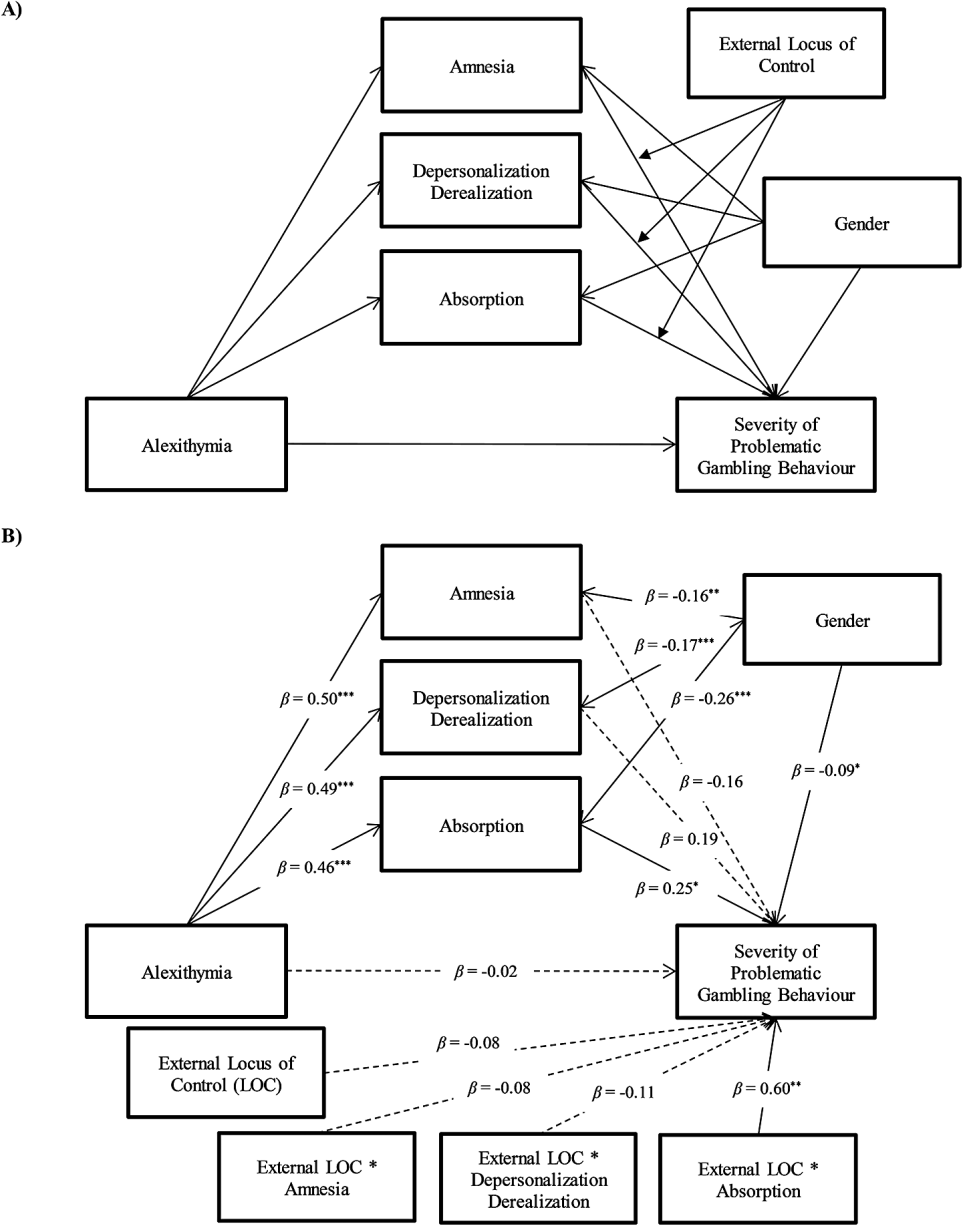
Statistical (**A**) and conceptual (**B**) forms of the moderated mediation model. *Note* *** *p <* 0.001; ** *p* < 0.01; * *p* < 0.05

A significant and positive total effect was found in the relationship between alexithymia and severity of problematic gambling behaviour (*β* = 0.38, *p* < 0.001), controlling for gender (*β* = -0.23, *p* < 0.001). Alexithymia was also significantly and positively associated with Amnesia (*β* = 0.50, *p* < 0.001), Depersonalization/Derealization (*β* = 0.49, *p* < 0.001) and Absorption (*β* = 0.46, *p* < 0.001). Only the latter, in turn, exhibits a significant and positive relationship with the severity of problematic gambling behaviour (*β* = 0.25, *p* < 0.05). Furthermore, External Locus of Control significantly moderated the association between Absorption and severity of problematic gambling behaviour: *ΔR*^2^ = 0.015, *F*(1, 280) = 9.725, *p* < 0.01 (see Fig. 1 part B). No other significant moderations by External Locus of Control were identified, neither in the direct path from alexithymia to severity of problematic gambling, nor in the indirect one involving Absorption.

**Fig. 2:**
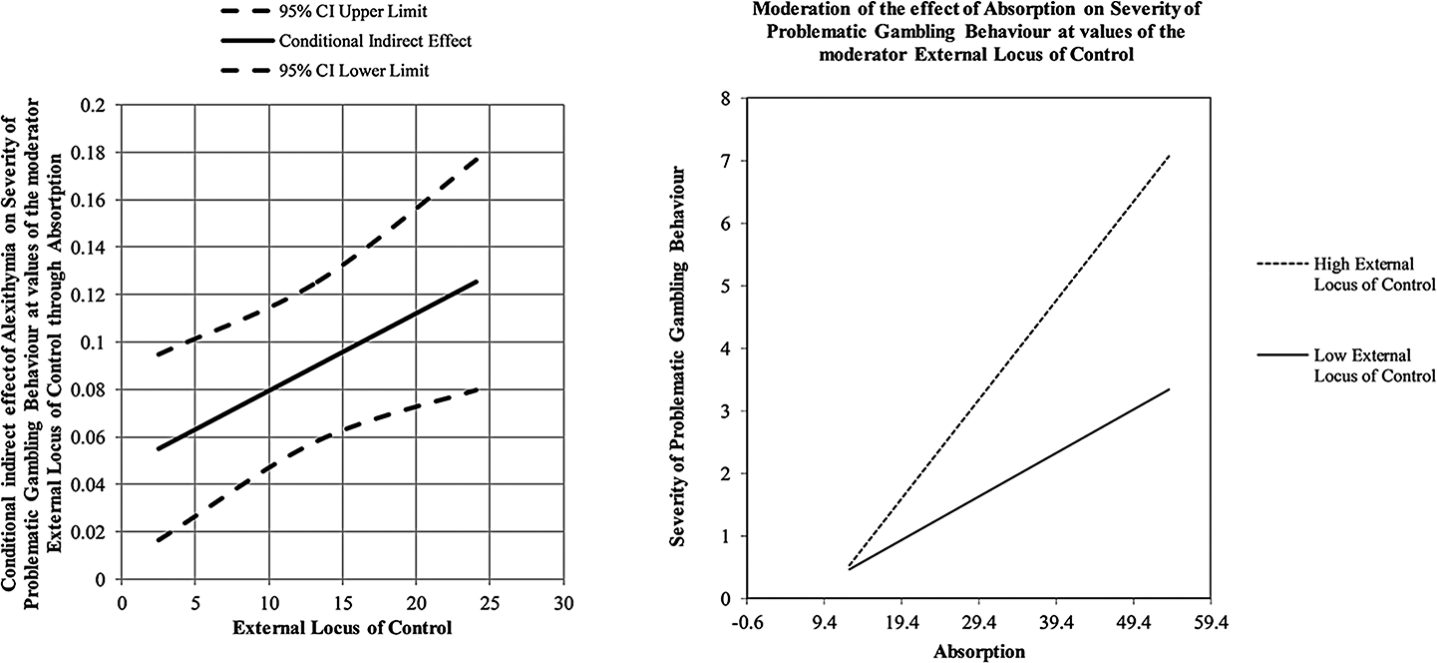
Graphic representation of the moderated-moderation effect

The conditional indirect effects of alexithymia on the severity of problematic gambling behaviour were also explored at three levels of the moderator (-1 SD, mean, + 1 SD): the effect was significant with an increasing trend as the external locus of control levels increased (see Table 3; Fig. 2).

**Table 3:** Unstandardized Coefficients of the model

	Effect	SE	BootLLCI	BootULCI
*Total Effect*	0.149	0.024	0.1106	0.1891
*Direct Effect*	0.001	0.021	− 0.0404	0.0441
*Conditional Indirect Effect at the values of the moderators*				
− 1SD	0.055	0.024	0.0167	0.0946
Mean	0.090	0.021	0.0584	0.1253
+ 1SD	0.125	0.030	0.0798	0.1769

Concerning the exploration of the covariate effects, being female was negatively associated with the dissociation subdimensions, (Amnesia, *β* = -0.16, *p* < 0.01; Depersonalization/Derealization, *β* = -0.17, *p* < 0.001; Absorption, *β* = 0.-26, *p* < 0.001), and with the severity of problematic gambling behaviour (*β* = -0.09, *p* < 0.05). Within the tested model, the direct effect in the relationship between alexithymia and severity of problematic gambling behaviour was non-significant (*β* = -0.02, *p* < 0.013), suggesting a fully moderated mediation explaining the 56% of the variance (a substantial effect): *R*^*2*^ = 0.562, *F*(9, 280) = 40.108, *p* < 0.001. Finally, the bootstrapping procedure (5000 bootstrapped samples) confirmed the statistical stability of the model (see Table 3).

## Discussion

Gambling disorder and the associated harm have been recognized as a worldwide public health challenge, in light of the clinical significance of this phenomenon (Abbott, 2020). Consequently, the international scientific literature on this condition is continually evolving and expanding, with the aim of understanding the potential underlying causes (e.g., see Moreira et al., 2023, for a review) and promoting the development of increasingly effective treatments (e.g., see Ribeiro et al., 2021, for a review). Given this framework, the present research aimed at exploring the association between some antecedent factors for gambling disorder, specifically investigating the role of alexithymia, dissociation, and locus of control.

First, gamblers with higher severity of problematic gambling behaviour showed significantly higher levels of alexithymia, dissociation, and external locus of control, confirming the first hypothesis (**H1**). This result is consistent with existing evidence. Indeed, previous research has found that individuals with gambling disorder exhibit higher levels of alexithymia (Bibby & Ross, 2017; Gori et al., 2016) and dissociation (Imperatori et al., 2017). Placed within a broader framework, these data support the possibility that these dimensions are involved in the development or maintenance of gambling disorders (see Marchetti et al., 2019; Schluter & Hodgins, 2019, for reviews), as well as behavioural addictions (e.g., Gori et al., 2021; Gori & Topino, 2023), in a more extensive view. Regarding locus of control, significant differences were found only in external LOC and not in internal LOC. In this regard, there is an ongoing debate in the scientific literature regarding the possibility of considering locus of control as a unidimensional or bidimensional construct (Clarke, 2004; Rotter, 1966). The findings of this and other studies (Thrasher et al., 2011) support the latter perspective. In fact, significant differences were found only for the external LOC dimension, consistent with previous speculations that emphasize believing that one’s outcomes during gambling are caused by external forces facilitates the perception of a lack of control over one’s behaviours, predisposing individuals to excessive and problematic gambling (Shumlich et al., 2017; Zhou et al., 2012).

Such data were further explored in the moderated mediation model. In this regard, a significant and positive association between alexithymia and the severity of problematic gambling behaviour was found (supporting **H2**), with the mediation of absorption in this relationship (partially confirming **H3**). These findings align with previous research on behavioural addiction (Gori et al., 2022b; Rogier et al., 2022) and support the view of compulsive behaviour as an outcome of an escape and avoidance strategy from unregulated emotional experiences through a focus on (dysfunctional) external regulatory strategies (McCormick et al., 2012). Consistently, the significant role of the dissociation sub-dimension of absorption, and not the others, is in line with findings on “dark flow,” which allows players experiencing it to escape into a pleasant altered state of awareness, increasing the risk of developing addiction (Dixon et al., 2019). Furthermore, the results revealed a significant moderation effect of external locus of control, indicating that as this factor increased, the impact of absorption on the severity of problematic gambling behaviours also increased (supporting **H4**). This finding is consistent with research on problematic online gaming suggesting that the motivation for escapism is a significant predictor of the intention to engage in online gaming, and this association is stronger among individuals characterized by an external locus of control (Chang et al., 2018; Koo, 2009). Finally, the role of gender as a potential confounder was controlled in conducting the moderated–mediation model. Although the relationships analysed in the model remained significant, a notable effect was observed towards both dissociation and the severity of problematic gambling behaviours. Specifically, being male was associated with higher scores in these variables, in accordance with previous scientific research (De Pasquale et al., 2018; Calado & Griffiths, 2016).

A series of limitations in this study must be acknowledged. First, the cross-sectional design hinders the tracking of changes over time and understanding the causal direction of observed relationships. To overcome this limitation, future research could adopt a longitudinal design, allowing for the examination of relationships over time and better identification of causal dynamics. This approach would provide a more in-depth understanding of the nature and direction of influences among variables involved in the context of gambling disorder. Another limitation lies in the generalization of findings across various gambling subtypes without specifically addressing potential differences among specific types of gambling activities. Future research should adopt a more nuanced approach by considering specific subtypes of gambling activities (e.g., sports betting, casino gambling) to identify distinct risk factors and motivational patterns associated with each subtype. Finally, the use of self-report instruments in this study may be susceptible to response bias, social desirability, or recall inaccuracies, potentially impacting the accuracy and reliability of the gathered data. To address this limitation, future research could incorporate a multi-method approach, combining self-report measures with objective assessments or observational data. Additionally, utilizing ecological momentary assessment (EMA) techniques could provide real-time and context-specific data, reducing reliance on retrospective self-reports and enhancing the validity of the findings in the study of gambling disorder.

### Conclusions

Falling within the broader framework of scientific research that focuses on risk factors for gambling disorder, the present study provides a further contribution in this field by investigating the associations of alexithymia, dissociation, and external locus of control with severity of problematic gambling behaviour in a sample of gambler. The results showed significant differences in these factors based on levels of Gambling Disease (Absence of Gambling Disease; At Risk for Gambling Disease; Problematic Gambling). Furthermore, the dissociation’s subdimension of absorption significantly mediated the relationship between alexithymia and the severity of problematic gambling behaviour. Finally, the external locus of control significantly moderated this indirect effect. Such data support the possibility that emotional dysregulation and dysfunctional tendencies toward avoidance for managing negative affects may be vulnerability factors for the development of problematic gambling (Rogier et al., 2021, 2022), especially in those who tend to perceive little control over the outcomes of their actions. These findings may have practical implications for clinical practice and prevention initiatives. Indeed, interventions focused on emotional regulation have already demonstrated their efficacy in the treatment of gambling disorder (Månsson et al., 2022). In addition to these, the results of the present research suggest the possibility of working specifically on the dissociative features associated with absorption, as well as on the external locus of control, to modify and mitigate dysfunctional strategies related to escapism.

## Electronic Supplementary Material

Below is the link to the electronic supplementary material.

Supplementary Material 1

## Data Availability

The data presented in this study are available on request from the corresponding author.
