# Supplementary material for: Problematic Gambling Behavior in a Sample of Gamblers: The Role of Alexithymia, Dissociation Features, and External Locus of Control
Source: J Gambl Stud. 2024 Jun 4;40(4):2077–91. doi: 10.1007/s10899-024-10322-6 (PMC11557621; doi:10.1007/s10899-024-10322-6)
Supplement: Supplementary file 1 — Supplementary Material 1 [file 10899_2024_10322_MOESM1_ESM.pdf]

Declaration of interests

☒ The authors declare that they have no known competing financial interests or personal relationships that could have appeared to influence the work reported in this paper.

☐The authors declare the following financial interests/personal relationships which may be considered as potential competing interests:
